# Supplementary material for: A Critical View of the Application of the APEX Software (Aqueous Photochemistry of Environmentally-Occurring Xenobiotics) to Predict Photoreaction Kinetics in Surface Freshwaters
Source: Molecules. 2019 Dec 18;25(1):9. doi: 10.3390/molecules25010009 (PMC7017383; doi:10.3390/molecules25010009)
Supplement: Supplementary file 1 [file molecules-25-00009-s001.zip › 3.2.4_gcc-4.4.0.zip!/3.2.4_gcc-4.4.0/3.2.4_gcc-4.4.0/doc/octave/html/interpreter/Cell-Arrays-of-Strings.html]

Cell Arrays of Strings - Untitled


Next: Processing Data in Cell Arrays,
Previous: Indexing Cell Arrays,
Up: Cell Arrays


---

#### 6.2.4 Cell Arrays of Strings

One common use of cell arrays is to store multiple strings in the same
variable. It is also possible to store multiple strings in a
character matrix by letting each row be a string. This, however,
introduces the problem that all strings must be of equal length.
Therefore, it is recommended to use cell arrays to store multiple
strings. For cases, where the character matrix representation is required
for an operation, there are several functions that convert a cell
array of strings to a character array and back. `char` and
`strvcat` convert cell arrays to a character array
(see Concatenating Strings), while the function `cellstr`
converts a character array to a cell array of strings:

```
     a = ["hello"; "world"];
     c = cellstr (a)
          => c =
              {
                [1,1] = hello
                [2,1] = world
              }
```

— Built-in Function: **cellstr** (string)  
> Create a new cell array object from the elements of the string
> array string.

One further advantage of using cell arrays to store multiple strings is
that most functions for string manipulations included with Octave
support this representation. As an example, it is possible to compare
one string with many others using the `strcmp` function. If one of
the arguments to this function is a string and the other is a cell array
of strings, each element of the cell array will be compared to the string
argument:

```
     c = {"hello", "world"};
     strcmp ("hello", c)
          => ans =
             1   0
```

The following string functions support cell arrays of strings:
`char`, `strvcat`, `strcat` (see Concatenating Strings), `strcmp`, `strncmp`, `strcmpi`,
`strncmpi` (see Comparing Strings), `str2double`,
`deblank`, `strtrim`, `strtrunc`, `strfind`,
`strmatch`, , `regexp`, `regexpi` (see Manipulating Strings) and `str2double` (see String Conversions).

The function `iscellstr` can be used to test if an object is a
cell array of strings.

— Built-in Function: **iscellstr** (cell)  
> Return true if every element of the cell array cell is a
> character string

— Function File: [idxvec, errmsg] = **cellidx** (listvar, strlist)  
> Return indices of string entries in listvar that match strings
> in strlist.
>
> Both listvar and strlist may be passed as strings or
> string matrices. If they are passed as string matrices, each entry
> is processed by `deblank` prior to searching for the entries.
>
> The first output is the vector of indices in listvar.
>
> If strlist contains a string not in listvar, then
> an error message is returned in errmsg. If only one output
> argument is requested, then cellidx prints errmsg to the
> screen and exits with an error.
